# Nano-Encapsulated Phytosterols Ameliorate Hypercholesterolemia in Mice via Dual Modulation of Cholesterol Metabolism Pathways

**DOI:** 10.3390/nu17132086

**Published:** 2025-06-23

**Authors:** Aixia Zhu, Wenjing Pan, Wenjia Jiao, Kai Peng, Chunwei Wang, Chi Zhang, Jiaqi Zhang

**Affiliations:** 1 Hubei Key Laboratory of Animal Nutrition and Feed Science, Wuhan Polytechnic University, Wuhan 430023, China; slgcx@whpu.edu.cn (A.Z.); 18652926613@163.com (W.P.); wenjia20131124@163.com (W.J.); wangchunwei605@163.com (C.W.); zhch@whpu.edu.cn (C.Z.); 2 Institute of Animal Science, Guangdong Academy of Agricultural Sciences, Key Laboratory of Animal Nutrition and Feed Science in South China, Ministry of Agriculture and Rural Affairs, Guangdong Provincial Key Laboratory of Animal Breeding and Nutrition, Guangzhou 510640, China; pengkai1016@126.com

**Keywords:** phytosterol nanoparticles, cholesterol regulation, CYP7A1, high-fat diet, mouse

## Abstract

**Background:** The limited bioavailability of free phytosterols restricts their clinical application in managing hypercholesterolemia. This study aimed to develop phytosterol nanoparticles (PNs) to enhance bioactivity and investigate their cholesterol-lowering efficacy and underlying mechanisms in vivo. **Methods:** Phytosterol nanoparticles (PNs) (93.35 nm) were engineered using soy protein isolate and administered orally at concentrations of 4.00–12.50 mg/mL to high-fat-diet-induced hypercholesterolemic mice (*n* = 60) over a 4-week period. Serum and hepatic lipid profiles, histopathology, gene/protein expression related to cholesterol metabolism, and fecal sterol content were evaluated. **Results:** PNs dose-dependently reduced serum total cholesterol (TC: 28.6–36.8%), triglycerides (TG: 22.4–30.1%), and LDL-C (31.2–39.5%), while increasing HDL-C by 18.7–23.4% compared to hyperlipidemic controls (*p* < 0.01). Hepatic TC and TG accumulation decreased by 34.2% and 41.7%, respectively, at the highest dose, with histopathology confirming attenuated fatty degeneration. Mechanistically, PNs simultaneously suppressed cholesterol synthesis through downregulating HMGCR (3.2-fold) and SREBP2 (2.8-fold), while enhancing cholesterol catabolism via CYP7A1 upregulation (2.1-fold) at protein level. Although less potent than simvastatin (*p* < 0.05), the nanoparticles exhibited unique dual-pathway modulation absent in conventional phytosterol formulations. Fecal analysis revealed dose-responsive cholesterol excretion (36.01 vs. 11.79 mg/g in controls), indicating enhanced enteric elimination. While slightly less potent than simvastatin (*p* < 0.05), PNs offered unique dual-pathway modulation absent in conventional phytosterol formulations. **Conclusions:** Nano-encapsulation significantly improves the bioavailability and hypocholesterolemic efficacy of phytosterols. PNs represent a promising nutraceutical strategy for cholesterol management by concurrently regulating cholesterol synthesis and catabolism, with potential application in both preventive and therapeutic contexts.

## 1. Introduction

Hypercholesterolemia remains a critical risk factor for cardiovascular diseases, affecting approximately 40% of the global adult population [1]. While pharmacological interventions like statins are effective, their long-term use is associated with adverse effects including hepatic dysfunction and myopathy [2,3]. Phytosterols, plant-derived structural analogs of cholesterol, have emerged as promising natural alternatives by competitively inhibiting intestinal cholesterol absorption [4]. However, their therapeutic potential is severely limited by poor aqueous solubility (<0.01% *w*/*w*) and low bioavailability (<5% in free form), bottlenecks that conventional formulations fail to address [5,6].

Current strategies to enhance phytosterol bioavailability focus on esterification or emulsification, yet these approaches exhibit inconsistent efficacy in vivo [7]. For instance, Zhang et al. (2011) reported only 7–10% reduction in LDL-C using esterified phytosterols in rodent models, while our preliminary studies revealed that unmodified phytosterols lose >60% bioactivity during gastrointestinal transit [8]. Crucially, prior research has predominantly focused on single-pathway modulation (e.g., HMGCR inhibition), neglecting the synergistic potential of simultaneously targeting cholesterol synthesis and catabolism [9]. Furthermore, while nanoparticle delivery systems have revolutionized drug delivery for anticancer agents (e.g., Paclitaxel albumin-bound nanoparticles) [9], their application in phytosterol formulations remains underexplored, particularly in animal models of metabolic dysfunction.

This study pioneers an innovation: (1) engineering phytosterol nanoparticles (PNs) using soy protein isolate to overcome solubility limitations, and (2) investigating concurrent regulation of HMGCR/SREBP2 (synthesis) and CYP7A1 (catabolism) pathways. Unlike previous studies using passive cholesterol-lowering mechanisms, our PN design leverages structural mimicry of chylomicrons to actively compete for intestinal uptake sites, while enabling sustained hepatic delivery. We hypothesize that nano-encapsulation will amplify phytosterol bioactivity through dose-dependent modulation of cholesterol homeostasis, a mechanism untested in prior formulations. Using a high-fat diet (HFD)-induced hypercholesterolemic ICR mouse model—a well-established system for mimicking human dyslipidemia—we systematically evaluate three NP concentrations (4.00–12.50 mg/mL) against simvastatin, the clinical gold standard. Our work addresses a critical gap in nutraceutical research by providing mechanistic evidence from gene expression (RT-qPCR) to protein-level validation (Western blot), setting a precedent for nanoparticle applications in veterinary medicine and functional feed development.

## 2. Materials and Methods

### 2.1. PN Preparation

PNs were prepared following the method described by Li et al. (2022) [10,11], with minor modifications. An aqueous solution of soy protein isolate (Fonterra Co-operative Group Ltd., Auckland, New Zealand) was prepared at a concentration of 0.75% (*w*/*w*) and allowed to hydrate overnight at 4 °C. In parallel, plant sterols (Wuhan Yuancheng Gongchuang Technology Co., Ltd., Wuhan, China) and lecithin (Shanghai Aladdin Bio-Chem Technology Co., Ltd., Shanghai, China) were dissolved in 16 mL of anhydrous ethanol at a weight ratio of 1:4 and stirred at 25 °C to form the organic phase.

The soy protein solution was pre-dispersed at 10,000× *g* and then combined with the organic phase. This mixture was subjected to high-speed homogenization for 5 min. The resulting emulsion was subsequently homogenized using a high-pressure homogenizer at 900 bar for six cycles, forming a stable nanodispersion. Ethanol was then removed via rotary evaporation to yield the final phytosterol nanoparticle formulation. The nanoparticles were diluted with deionized water to achieve final phytosterol concentrations of 4.00, 8.25, and 12.50 mg/mL. The resulting nanoparticles had an average particle size of 93.35 nm, a zeta potential of −29.3 mV, and an encapsulation efficiency of 97.26%, as characterized using a Turbiscan stability analyzer (Formulaction, Toulouse, France). Their long-term physical stability, including changes in particle size, zeta potential, and dispersion behavior under storage at 4 °C and room temperature for up to 30 days, was confirmed in our previous study [10], showing minimal variation (zeta potential −29.3 mV ± 1.2) and no significant aggregation or degradation.

### 2.2. Diet Preparation, Experimental Design, and Sample Collection

All experimental protocols were approved by the Hubei Provincial Department of Science and Technology, China. The animal study was conducted at the Laboratory Animal Center of Huazhong University of Science and Technology, which holds a Laboratory Animal Production License [SCXK (Hubei) 2016-20009] and a Laboratory Animal Use License [SYXK (Hubei) 2016-0057]. Specific pathogen-free (SPF) grade male ICR mice (4 weeks old) were obtained from the Hubei Provincial Experimental Animal Center. After the one-week acclimatization period, mice were randomly assigned to experimental groups using a computer-generated randomization schedule. A total of 60 mice were randomly assigned to six treatment groups (*n* = 10 per group), with five replicate cages per group. The sample size of 10 mice per group was determined based on prior studies utilizing comparable hyperlipidemia models and evaluating similar outcome measures, such as serum lipid profiles and hepatic gene/protein expression. In those studies, group sizes ranging from 8 to 12 animals were sufficient to detect statistically significant differences with acceptable variability and statistical power. Therefore, a group size of *n* = 10 was deemed appropriate to ensure reliable and reproducible results in this experimental context.

The specific experimental groupings are detailed in Table 1. During the initial four-week induction phase, mice were fed either a standard chow diet (Normal group, Nor) or a high-fat diet (HFD) to induce hypercholesterolemia. Subsequently, over a four-week intervention period, HFD-fed mice received one of the following daily oral gavage treatments (0.5 mL per dose): PNs at concentrations of 4.00 mg/mL (L-PN), 8.25 mg/mL (M-PN), or 12.50 mg/mL (H-PN); simvastatin at 0.40 mg/mL (PM, positive control); or normal saline (HCM, model control). The composition and nutritional profiles of the diets are detailed in Table 2. Adlibitum intake and water intake during the trial. The phytosterol nanoparticle concentrations (4.00, 8.25, and 12.50 mg/mL) were selected based on preliminary pilot studies in mice, which indicated good biocompatibility, no observable toxicity, and stable dispersion properties up to 12.50 mg/mL. Additionally, the selected doses corresponded to an approximate intake range of 20–60 mg/kg body weight per day, which aligns with effective dose ranges reported in the literature for phytosterols in rodent models of hyperlipidemia [8,10]. Doses exceeding 12.50 mg/mL resulted in reduced nanoparticle stability and were thus excluded from further testing.

Throughout the study, animals were housed in a controlled environment (temperature: 22–25 °C; relative humidity: 55–70%) with a 12 h light/dark cycle and had free access to food and water. Body weight was recorded weekly, and feed intake was measured every three days.

Fecal samples were collected from each group three days prior to and at the conclusion of the treatment period. Samples were freeze-dried under vacuum, sealed, and stored at −80 °C for further analysis. At the end of the experiment, mice were anesthetized with isoflurane, and blood samples were collected via orbital sinus puncture; plasma was separated by centrifugation (900× *g*, 15 min, 4 °C) and stored at −80 °C. Mice were then euthanized by cervical dislocation. Livers were immediately excised, rinsed with sterile 0.9% saline, blotted dry, and weighed: a portion (0.2 g) was flash-frozen in liquid nitrogen for RT-PCR and Western Blot analysis, another portion (1.0 ± 0.05 g) was wrapped in aluminum foil for lipid quantification (stored at −20 °C), and the remaining tissue was fixed in neutral-buffered formalin for histological examination. During sample collection and subsequent analyses, all samples were labeled with animal identification numbers. Although these IDs were visible to the personnel conducting biochemical and molecular assays, the treatment group assignments were not indicated, and all analyses were conducted following standardized procedures to reduce potential bias.

### 2.3. Biochemical and Histological Assessments

#### 2.3.1. Plasma Lipid Profile

Plasma concentrations of total cholesterol (TC), triglycerides (TG), high-density lipoprotein cholesterol (HDL-C), and low-density lipoprotein cholesterol (LDL-C) were determined using commercial enzymatic assay kits (Nanjing Jiancheng Bioengineering Institute, Nanjing, China), following the manufacturer’s instructions.

#### 2.3.2. Hepatic Lipid Content

Approximately 1.0 g of liver tissue was homogenized in a chloroform/methanol solution (2:1, *v*/*v*) on ice. After filtration and triple rinsing with 0.2 volumes of the same solvent, the total filtrate was adjusted to 25 mL. Hepatic TC and TG levels were quantified using an automated biochemical analyzer.

#### 2.3.3. Fecal Sterol and Cholesterol Analysis

Fecal samples (~0.05 g) were extracted with 5 mL of chloroform/methanol (1:1, *v*/*v*) using ultrasonic treatment for 5 min, followed by incubation in a 60 °C water bath for 18 h. After centrifugation, 2.5 mL of the supernatant was transferred and mixed with 400 μL of 5α-cholestane as an internal standard. Saponification was performed using 10 mL of 1 mol/L ethanolic KOH at 80 °C for 1 h. After extraction with water and *n*-hexane, and triple washing, the organic phase was dried under nitrogen. The residue was derivatized with 0.1 mL pyridine and 0.1 mL BSTFA/TMCS at 70 °C for 1 h. Final samples were analyzed by gas chromatography (GC) on an HP-5 capillary column (30 m × 0.32 mm × 0.25 μm) under a programmed temperature gradient. The carrier gas flow rate was 1 mL/min with a split ratio of 10:1.

#### 2.3.4. Liver Histology

Liver tissue samples were fixed in 10% neutral-buffered formalin, embedded in paraffin, sectioned at 4 μm thickness, and stained with hematoxylin and eosin (H&E). Histological observations were made under a light microscope, and images were analyzed using Leica Application Suite software (version 4.15, Leica Microsystems, Wetzlar, Germany).

#### 2.3.5. Gene Expression Analysis (RT-qPCR)

Total RNA was isolated from liver tissue using Trizol reagent. RNA quality and concentration were assessed via 1% agarose gel electrophoresis and UV spectrophotometry (Evolution 220, Thermo Fisher, Waltham, MA, USA). Reverse transcription to cDNA and real-time quantitative PCR (RT-qPCR) were conducted using specific primers (Table 3). The amplification protocol included an initial denaturation at 95 °C for 3 min, followed by 39 cycles of 95 °C for 5 s, 56 °C for 10 s, and 72 °C for 25 s, concluding with a final extension at 95 °C for 50 s.

#### 2.3.6. Protein Expression Analysis (Western Blot)

Total liver proteins were extracted and quantified using a BCA protein assay kit (Bioswamp, Wuhan, China) [12,13]. Equal amounts of protein were separated by SDS-PAGE, transferred to PVDF membranes, and blocked with 5% non-fat milk. Membranes were incubated with primary antibodies against HMGCR, SREBP2, CYP7A1, LDLR, and LXR-α (Abcam, Cambridge, UK), followed by HRP-conjugated secondary antibodies. Protein bands were detected using enhanced chemiluminescence (ECL) and quantified with TANON GIS image analysis software (version 4.2.5, Tanon Science & Technology, Shanghai, China).

### 2.4. Data Calculations and Statistical Analysis

All results are presented as mean ± standard deviation (SD), based on three independent replicates per treatment group. Statistical analyses were performed using SPSS version 19.0 (IBM Corp., Armonk, NY, USA). Differences between two groups were assessed using independent-samples *t*-tests, while multiple group comparisons were analyzed using one-way analysis of variance (ANOVA), followed by Duncan’s multiple range test for post hoc analysis if variances were homogeneous. In cases where the assumption of homogeneity of variances was violated, non-parametric tests (e.g., Kruskal–Wallis test) were used instead. A *p*-value of less than 0.05 was considered statistically significant, and *p* < 0.01 was regarded as highly significant. Statistical significance levels are indicated in the figures and tables as *p* < 0.05 (*) and *p* < 0.01 (**). Data visualization, including error bars, was performed using GraphPad Prism 8.0 (GraphPad Software, San Diego, CA, USA).

## 3. Results

### 3.1. Average Body Weight and Daily Feed Intake

**Figure 1:**
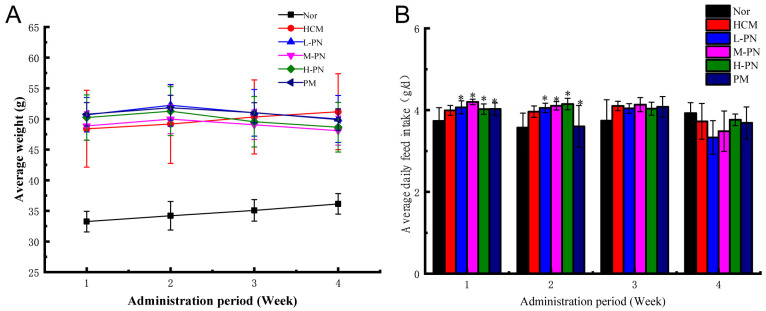
Average body weight (**A**) and average daily feed intake (**B**) of mice. Nor: fed basic feed + 0.5 mL saline gavage daily; HCM: fed high-fat feed + 0.5 mL saline gavage daily; L-PN: fed high-fat feed + 0.5 mL low concentration (4.00 mg/mL) PNs by gavage daily; M-PN: fed high-fat feed + 0.5 mL medium concentration (8.25 mg/mL) PNs by gavage daily; H-PN: fed high-fat diet + 0.5 mL high concentration (12.50 mg/mL) PNs by daily gavage; PM: fed high-fat diet + 0.5 mL 0.4 mg/mL simvastatin by daily gavage. * (*p* < 0.05) vs. Nor. *n* = 10.

Throughout the experimental period, the body weight of mice in all groups was monitored, and the average daily feed intake was calculated (Figure 1). After four weeks of treatment, mice in the phytosterol nanoparticle groups (L-PN, M-PN, and H-PN) and the simvastatin group (PM) exhibited lower average body weights compared to the hypercholesterolemic model group (HCM) (Figure 1A). This trend suggests a potential effect of both PNs and simvastatin in mitigating body weight gain induced by a high-fat diet. Notably, there was no clear dose-dependent reduction in body weight among the phytosterol-treated groups within the tested concentration range.

In terms of daily feed intake, no significant differences were observed between the phytosterol nanoparticle groups (L-PN, M-PN, and H-PN) and the hypercholesterolemia model group (HCM) compared with PM (*p* > 0.05), suggesting that neither the PNs nor simvastatin affected appetite or food consumption during the study period (Figure 1B). These results suggest that the observed differences in body weight may be due to metabolic regulation rather than changes in energy intake.

### 3.2. Serum and Liver Lipid Profile

**Figure 2:**
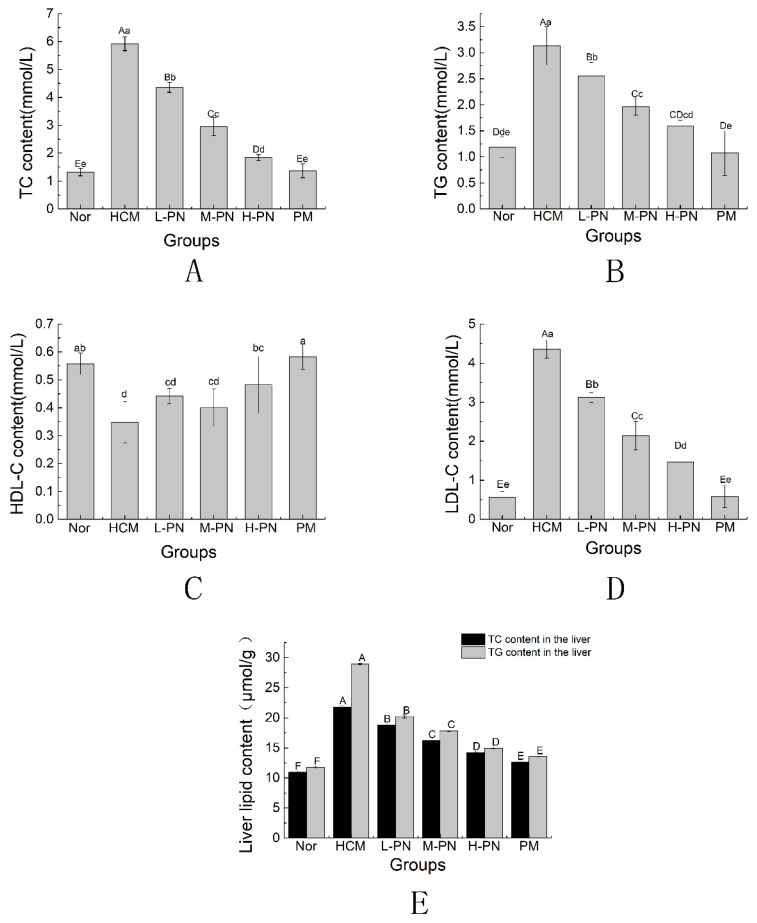
Serum TC (**A**), TG (**B**), HLD-C (**C**), LDL-C (**D**) and liver lipid content (**E**) concentrations. Nor: fed basic feed + 0.5 mL saline gavage daily; HCM: fed high-fat feed + 0.5 mL saline gavage daily; L-PN: fed high-fat feed + 0.5 mL low concentration (4.00 mg/mL) PNs by gavage daily; M-PN: fed high-fat feed + 0.5 mL medium concentration (8.25 mg/mL) PNs by gavage daily; H-PN: fed high-fat diet + 0.5 mL high concentration (12.50 mg/mL) PNs by daily gavage; PM: fed high-fat diet + 0.5 mL 0.4 mg/mL simvastatin by daily gavage. Different capital letters and lowercase letters indicate highly significant (*p* < 0.01) and significant (*p* < 0.05) differences between groups. *n* = 10.

Serum concentrations of total cholesterol (TC), triglycerides (TG), high-density lipoprotein cholesterol (HDL-C), and low-density lipoprotein cholesterol (LDL-C) are shown in Figure 2. At the end of the experimental period, mice in the hypercholesterolemic model group (HCM) exhibited significantly elevated levels of serum TC, TG, and LDL-C, along with a marked decrease in HDL-C, compared to the normal control group (Nor) (*p* < 0.01). These results confirm the successful establishment of a hyperlipidemic model.

Oral administration of PNs (L-PN, M-PN, H-PN) and simvastatin (PM) for four weeks significantly reduced serum TC, TG, and LDL-C concentrations compared to the HCM group (*p* < 0.01). Additionally, the H-PN and PM groups demonstrated significant increases in HDL-C levels compared to the model group (*p* < 0.05), restoring them to near-normal levels. A dose-dependent trend was observed among the phytosterol-treated groups, with higher concentrations generally producing greater lipid-lowering effects.

In parallel, hepatic lipid analysis revealed that the HCM group had markedly elevated levels of TC and TG in liver tissue, showing increases of 99.04% and 145.95%, respectively, compared to the Nor group (*p* < 0.01). Treatment with PNs significantly attenuated hepatic lipid accumulation in a dose-dependent manner, with the H-PN group exhibiting the most pronounced reductions (*p* < 0.01). Simvastatin (PM) produced comparable improvements. These results suggest that PNs effectively ameliorate both systemic and hepatic lipid disorders induced by a high-fat diet.

### 3.3. Liver Histomorphology

**Figure 3:**
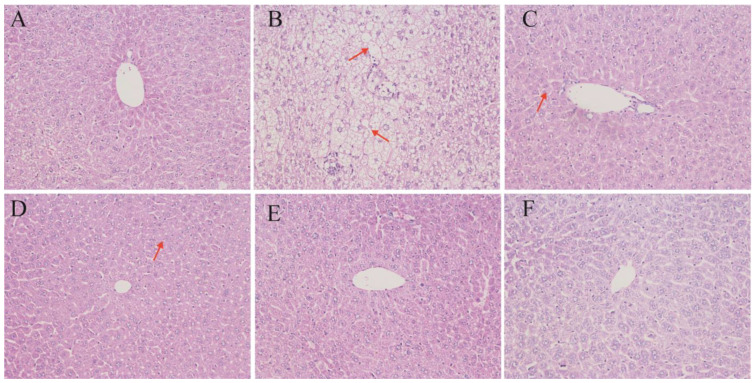
Liver histomorphology (×200). Nor: fed basic feed + 0.5 mL saline gavage daily (**A**); HCM: fed high-fat feed + 0.5 mL saline gavage daily (**B**); L-PN: fed high-fat feed + 0.5 mL low concentration (4.00 mg/mL) PNs by gavage daily (**C**); M-PN: fed high-fat feed + 0.5 mL medium concentration (8.25 mg/mL) PNs by gavage daily (**D**); H-PN: fed high-fat diet + 0.5 mL high concentration (12.50 mg/mL) PNs by daily gavage (**E**); PM: fed high-fat diet + 0.5 mL 0.4 mg/mL simvastatin by daily gavage (**F**). Red arrows indicate representative hepatocytes exhibiting cytoplasmic vacuolization or lipid droplets, characteristic of hepatic steatosis.

To visually assess the effects of PNs on liver morphology, histomorphology analysis of hepatic tissue was performed (Figure 3). Histological examination of liver tissues revealed substantial structural alterations in response to dietary and treatment interventions. In the normal control group (Nor), hepatocytes exhibited uniform morphology with tightly arranged cell cords and intact cellular architecture, indicative of healthy liver tissue. In contrast, liver sections from the hypercholesterolemic model group (HCM) displayed significant pathological changes, including hepatocellular swelling, disrupted cellular organization, and the presence of large vacuoles indicative of lipid accumulation and steatosis.

Treatment with simvastatin (PM) markedly improved hepatic morphology, as evidenced by well-preserved hepatocyte structure and reduced intracellular lipid vacuolation. Similarly, administration of PNs at low (L-PN), medium (M-PN), and high (H-PN) concentrations mitigated hepatic steatosis to varying degrees. Hepatocytes in these groups appeared more orderly, with diminished cytoplasmic vacuolization and less extensive tissue disruption compared to the HCM group. The H-PN group exhibited the most substantial histological improvement among the phytosterol-treated groups. These findings confirm that PNs effectively alleviate liver damage and lipid deposition induced by a high-fat diet, supporting their potential hepatoprotective role in hypercholesterolemic conditions.

To quantitatively assess hepatic histopathological changes, we applied the NAFLD Activity Score (NAS), a semi-quantitative scoring system that evaluates steatosis (0–3), lobular inflammation (0–3), and hepatocellular ballooning (0–2). As shown in Table 4, the HCM group exhibited the highest total NAS score (7), characterized by extensive macrovesicular steatosis, moderate inflammation, and marked ballooning. In contrast, the simvastatin-treated group (PM) and all phytosterol nanoparticle-treated groups showed significantly reduced scores. The high-dose phytosterol group (H-PN) demonstrated the greatest histological improvement, with NAS scores reduced to 2–3, indicating minimal steatosis and nearly normal hepatic architecture. These scoring results corroborate the qualitative observations and further confirm the hepatoprotective effect of PNs.

Scoring criteria were based on Kleiner et al. (2005) [14]: steatosis (0–3), lobular inflammation (0–3), and hepatocellular ballooning (0–2). Scores represent average observations from two independent, blinded pathologists.

### 3.4. Fecal Cholesterol and Phytosterol Excretion

The results of fecal cholesterol and sterol content are summarized in Table 5. Quantitative analysis of fecal samples revealed significant differences in cholesterol excretion among the experimental groups. Compared to the normal control group (Nor), the hypercholesterolemic model group (HCM) exhibited a marked increase in fecal cholesterol content (*p* < 0.01), likely due to impaired lipid metabolism and compensatory excretory mechanisms.

Treatment with PNs resulted in a dose-dependent elevation of fecal cholesterol levels. Specifically, mice in the L-PN, M-PN, and H-PN groups demonstrated significantly higher fecal cholesterol excretion than the HCM group (*p* < 0.01), with the H-PN group reaching the highest level of excretion (36.01 mg/g). The simvastatin-treated group (PM) showed a comparable increase in fecal cholesterol (36.90 mg/g), suggesting a similar enhancement of intestinal cholesterol elimination. In addition to cholesterol, detectable levels of phytosterols were identified in the feces of all phytosterol-treated groups, also in a dose-responsive manner. This finding indicates that part of the administered phytosterols were not absorbed and were directly excreted, which may contribute to their cholesterol-lowering effect via competitive inhibition of intestinal cholesterol absorption.

Collectively, these results suggest that PNs not only reduce cholesterol absorption in the gut but also enhance its fecal excretion, thereby contributing to improved cholesterol homeostasis.

### 3.5. Expression of Genes and Proteins Involved in Lipid Metabolism

**Figure 4:**
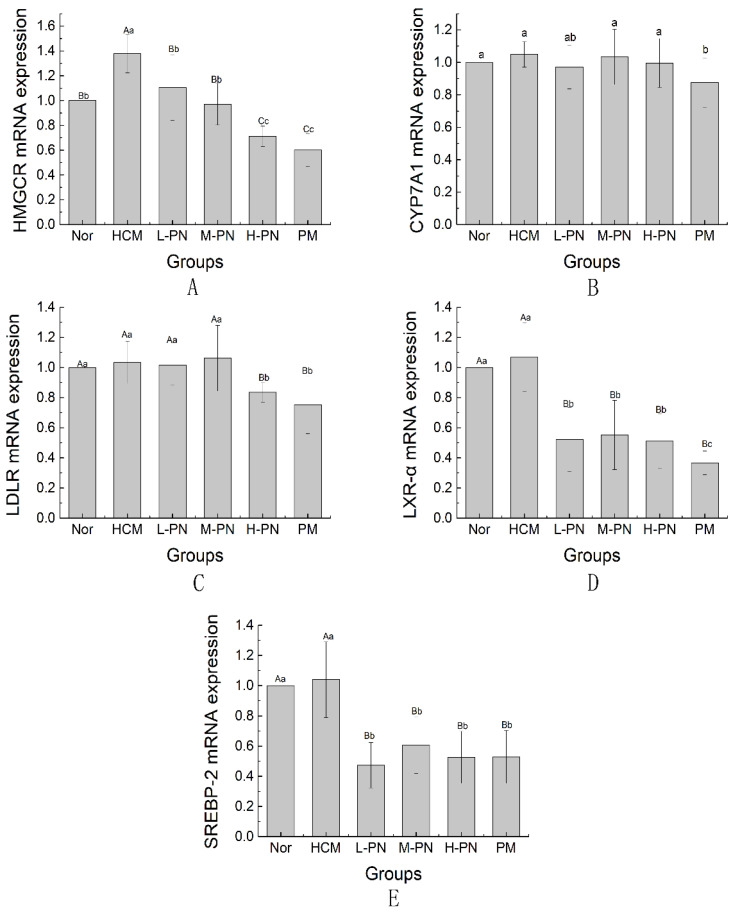
The mRNA expression of *HMGCR* (**A**)*, CYP7A1* (**B**), *LDLR* (**C**), *LXR-α* (**D**) and *SREBP 2* (**E**). Different capital letters and lowercase letters indicate highly significant (*p* < 0.01) and significant (*p* < 0.05) differences between groups. HMGCR, 3-Hydroxy-3-Methylglutaryl-CoA Reductase; SREBP2, Sterol Regulatory Element-Binding Protein 2; CYP7A1, Cholesterol 7α-Hydroxylase; LDLR, Low-Density Lipoprotein Receptor; LXR-α, Liver X Receptor Alpha; Nor: fed basic feed + 0.5 mL saline gavage daily; HCM: fed high-fat feed + 0.5 mL saline gavage daily; L-PN: fed high-fat feed + 0.5 mL low concentration (4.00 mg/mL) PNs by gavage daily; M-PN: fed high-fat feed + 0.5 mL medium concentration (8.25 mg/mL) PNs by gavage daily; H-PN: fed high-fat diet + 0.5 mL high concentration (12.50 mg/mL) PNs by daily gavage; PM: fed high-fat diet + 0.5 mL 0.4 mg/mL simvastatin by daily gavage. *n* = 10.

**Figure 5:**
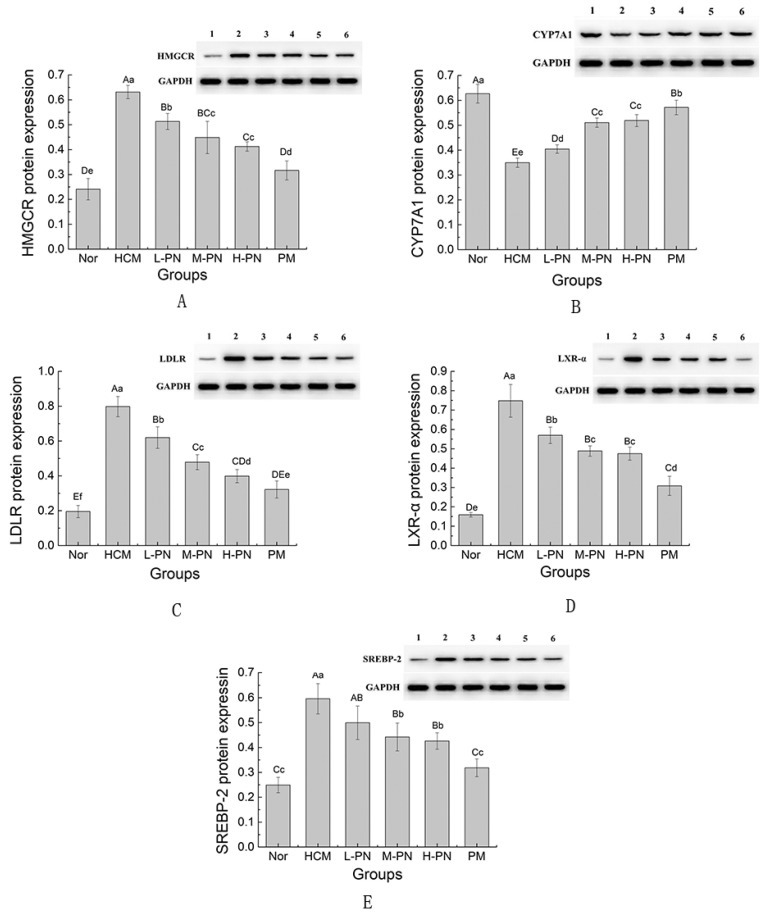
The protein expression of HMGCR (**A**), CYP7A1 (**B**), LDLR (**C**), LXR-α (**D**) and SREBP 2 (**E**). Different capital letters and lowercase letters indicate highly significant (*p* < 0.01) and significant (*p* < 0.05) differences between groups. HMGCR, 3-Hydroxy-3-Methylglutaryl-CoA Reductase; SREBP2, Sterol Regulatory Element-Binding Protein 2; CYP7A1, Cholesterol 7α-Hydroxylase; LDLR, Low-Density Lipoprotein Receptor; LXR-α, Liver X Receptor Alpha; Nor: fed basic feed + 0.5 mL saline gavage daily; HCM: fed high-fat feed + 0.5 mL saline gavage daily; L-PN: fed high-fat feed + 0.5 mL low concentration (4.00 mg/mL) PNs by gavage daily; M-PN: fed high-fat feed + 0.5 mL medium concentration (8.25 mg/mL) PNs by gavage daily; H-PN: fed high-fat diet + 0.5 mL high concentration (12.50 mg/mL) PNs by daily gavage; PM: fed high-fat diet + 0.5 mL 0.4 mg/mL simvastatin by daily gavage. *n* = 3. Numbers 1 to 6 correspond to the following groups: 1 = Nor, 2 = HCM, 3 = L-PN, 4 = M-PN, 5 = H-PN, 6 = PM.

To investigate the regulatory effects of PNs on cholesterol metabolism, the hepatic expression levels of key genes and their corresponding proteins—HMGCR, SREBP2, CYP7A1, LDLR, and LXR-α—were evaluated (Figure 4 and Figure 5). As the rate-limiting enzyme in cholesterol biosynthesis, HMGCR (3-Hydroxy-3-Methylglutaryl-CoA Reductase) was significantly upregulated at both the mRNA and protein levels in the HCM group compared to the normal control (*p* < 0.01), indicating enhanced endogenous cholesterol synthesis in hypercholesterolemic conditions (Figure 4A and Figure 5A). Treatment with PNs resulted in a dose-dependent downregulation of HMGCR expression. In the high-dose group (H-PN), HMGCR mRNA and protein levels were significantly reduced and were comparable to those observed in the simvastatin (PM) group (*p* < 0.01), suggesting that PNs effectively suppress cholesterol biosynthesis.

As the rate-limiting enzyme in bile acid synthesis, CYP7A1(Cholesterol 7α-Hydroxylase) plays a pivotal role in cholesterol catabolism. In the HCM group, CYP7A1 protein levels were markedly reduced compared to the Nor group (*p* < 0.01), indicating impaired cholesterol breakdown (Figure 4B and Figure 5B). While CYP7A1 mRNA expression did not differ significantly across groups, PNs significantly increased CYP7A1 protein levels in a dose-dependent manner (*p* < 0.01). This divergence between mRNA and protein expression may be due to post-transcriptional regulatory mechanisms or differential molecular stability. The highest expression of CYP7A1 protein was observed in the H-PN and PM groups, indicating enhanced bile acid synthesis and cholesterol clearance.

LDLR (Low-Density Lipoprotein Receptor) expression was elevated at both mRNA and protein levels in the HCM group, possibly reflecting a compensatory response to increased circulating LDL-C. Following treatment, PNs led to a moderate but significant reduction in LDLR protein expression (*p* < 0.01), particularly in the H-PN group (Figure 4C and Figure 5C). However, mRNA levels did not show a consistent pattern, suggesting that LDLR may be subject to complex post-transcriptional regulation in the context of cholesterol feedback.

SREBP2 (Sterol Regulatory Element-Binding Protein 2), a transcription factor that regulates the expression of HMGCR and other cholesterol synthesis genes, also showed significant upregulation in the HCM group at both transcriptional and translational levels (*p* < 0.01). Phytosterol nanoparticle administration significantly decreased SREBP2 mRNA and protein expression in a dose-dependent manner (*p* < 0.01) (Figure 4E and Figure 5E). These results imply that phytosterols may exert upstream regulatory effects on cholesterol biosynthesis by inhibiting SREBP2 activation.

As a nuclear receptor that regulates cholesterol efflux and CYP7A1 expression, LXR-α (Liver X Receptor Alpha) was significantly upregulated in the HCM group at both gene and protein levels. Administration of PNs significantly downregulated LXR-α expression, with the H-PN group showing the most pronounced suppression (*p* < 0.01). This suggests that phytosterol-mediated cholesterol reduction may also involve modulation of nuclear receptor activity.

Overall, PNs exhibited dual regulatory activity on cholesterol homeostasis by downregulating cholesterol synthesis pathways (HMGCR, SREBP2) and promoting cholesterol catabolism through enhanced CYP7A1 protein expression. The partial modulation of LDLR and LXR-α further supports the involvement of feedback regulatory mechanisms. These molecular findings are consistent with the observed physiological improvements in lipid profiles, reinforcing the potential of PNs as functional agents for cholesterol control.

## 4. Discussion

In this study, we successfully developed and evaluated a PN system with significantly enhanced cholesterol-lowering efficacy in a high-fat diet-induced hypercholesterolemic mouse model. Through comprehensive analyses including physiological monitoring, serum and hepatic lipid profiling, histopathological examination, fecal sterol quantification, and gene/protein expression studies, we demonstrated that PNs regulate cholesterol homeostasis through a dual-action mechanism: inhibition of cholesterol synthesis and promotion of cholesterol catabolism, supplemented by intestinal absorption blockade.

Simvastatin was included as a positive control due to its well-established lipid-lowering effect via HMG-CoA reductase inhibition. However, statin therapy may lead to adverse effects such as myopathy, elevated liver enzymes, or statin intolerance in some individuals [15]. In contrast, PNs represent a safer, dietary-derived alternative that may be particularly beneficial for statin-intolerant individuals or as a prophylactic intervention in early stage dyslipidemia. Unlike pharmacological statins, phytosterols act through both hepatic and intestinal pathways, potentially offering effective lipid regulation with fewer systemic side effects. Therefore, PNs may serve as a promising complementary or alternative strategy in lipid management, with strong translational potential.

### 4.1. Dual Modulation of Cholesterol Metabolism: Synthesis Suppression and Catabolism Enhancement

One of the most notable outcomes of this study is the simultaneous downregulation of HMGCR and SREBP2, and the upregulation of CYP7A1 protein expression in the liver. This dual modulation represents a significant advancement over traditional cholesterol-lowering strategies, which typically target only one pathway. HMGCR encodes 3-hydroxy-3-methylglutaryl-CoA reductase, the rate-limiting enzyme in cholesterol biosynthesis, and is a well-established pharmacological target of statins [16]. The suppression of both HMGCR gene and protein expression observed in all PN-treated groups—especially in the high-dose group—suggests that PNs effectively mimic the statin-like inhibition of endogenous cholesterol synthesis.

Moreover, SREBP2 (sterol regulatory element-binding protein 2) is a transcription factor that promotes the expression of HMGCR and other lipid biosynthesis genes [17]. The observed downregulation of SREBP2 further supports the hypothesis that PNs act upstream to attenuate cholesterol synthesis at the transcriptional regulatory level. This suppression of the SREBP2-HMGCR axis indicates a coordinated control mechanism that is likely triggered by increased cellular cholesterol levels resulting from reduced absorption and increased catabolism [18,19].

On the catabolic side, CYP7A1 encodes cholesterol 7α-hydroxylase, the rate-limiting enzyme for bile acid synthesis [7,20]. Interestingly, while CYP7A1 protein levels increased significantly in phytosterol-treated groups, mRNA levels did not show a corresponding elevation. This divergence may be attributed to post-transcriptional regulatory mechanisms such as enhanced protein stability, translational control, or regulation by microRNAs (miRNAs) [21]. Previous studies have shown that CYP7A1 protein expression can be influenced independently of its transcriptional activity, for example via miRNA-mediated suppression of mRNA translation or modulation of protein degradation pathways [22,23,24]. These findings suggest that PNs may act through such regulatory layers to increase CYP7A1 protein abundance and promote bile acid synthesis [25].

Together, the inhibition of cholesterol synthesis and the enhancement of its catabolism establish PNs as a dual-modality therapeutic, offering advantages over monofunctional treatments such as statins, ezetimibe, or bile acid sequestrants.

### 4.2. Intestinal Cholesterol Absorption Blockade and Fecal Sterol Excretion

Another critical finding is the increased fecal excretion of cholesterol and phytosterols in all PN-treated groups. Phytosterols are structurally similar to cholesterol and are known to compete for incorporation into mixed micelles in the intestinal lumen, displacing cholesterol and reducing its absorption [26]. This competitive inhibition occurs at the level of the Niemann-Pick C1-like 1 (NPC1L1) transporter. Previous reports have shown that phytosterols can significantly downregulate NPC1L1 expression and/or inhibit its function, thereby reducing cholesterol uptake by enterocytes [6,27].

One proposed mechanism for the inhibition of intestinal cholesterol absorption by phytosterols involves the Niemann–Pick C1-like 1 (NPC1L1) transporter, a key cholesterol uptake protein expressed on the apical surface of enterocytes [28]. Phytosterols are thought to compete with cholesterol for incorporation into mixed micelles, thereby limiting NPC1L1-mediated uptake. Although NPC1L1 expression was not directly measured in this study, the observed increase in fecal cholesterol excretion and reduction in serum lipid levels are consistent with decreased intestinal absorption, possibly involving inhibition of NPC1L1 activity [29]. Future studies will aim to quantify NPC1L1 expression to further confirm this mechanism.

Our results confirm that PNs significantly increase fecal cholesterol content in a dose-dependent manner. The concurrent detection of phytosterols in the feces suggests that not all of the administered sterols were absorbed, and their presence in the intestinal tract contributes to cholesterol displacement. These results are consistent with previous in vivo studies reporting enhanced sterol excretion following dietary phytosterol supplementation [30], but our findings indicate that nanoparticle encapsulation significantly amplifies this effect. This is likely due to increased dispersion and bioavailability, as nanoparticle formulations improve the solubilization and intestinal residence time of hydrophobic compounds such as phytosterols. The combined inhibition of absorption and stimulation of excretion provides a complementary mechanism to hepatic metabolic regulation, reinforcing the cholesterol-lowering efficacy of PNs.

### 4.3. Liver Protection and Histological Improvements

Histological analysis of liver sections revealed that PNs attenuated hepatic steatosis and preserved hepatocellular structure. The model group (HCM) showed typical features of fatty liver, including enlarged hepatocytes, cytoplasmic vacuolization, and disrupted architecture. In contrast, PN-treated mice exhibited dose-dependent improvements in tissue morphology, comparable to the simvastatin group. These improvements are attributed to reductions in hepatic lipid accumulation and restored lipid metabolism. As the liver plays a central role in cholesterol homeostasis, maintaining hepatic integrity is essential for systemic lipid balance [31,32].

These histological findings validate the biochemical results and provide morphological evidence of the therapeutic potential of PNs. In addition to their systemic lipid-lowering effects, the nanoparticles also offer localized hepatic protection, which may be beneficial in preventing non-alcoholic fatty liver disease (NAFLD) associated with dyslipidemia.

### 4.4. Comparative Effectiveness and Advantages over Simvastatin

While simvastatin, a widely used statin, showed slightly stronger inhibition of HMGCR, PNs achieved broader regulatory coverage by also modulating CYP7A1 and enhancing cholesterol excretion. Furthermore, phytosterols are naturally derived compounds with a long history of dietary use and minimal adverse effects [33,34]. In contrast, statins, though effective, are associated with risks such as hepatic dysfunction, myopathy, and increased diabetes incidence with long-term use [35,36]. The nanoparticle delivery system enhances the solubility and absorption of phytosterols, addressing the long-standing challenge of their poor bioavailability in native form [37]. Thus, PNs may serve as a safe, well-tolerated, and multifunctional alternative to statins, particularly for populations with statin intolerance or those seeking plant-based, non-pharmacological interventions.

### 4.5. Role of LDLR and LXR-α: Feedback Regulation and Complexity

The expression patterns of LDLR (low-density lipoprotein receptor) and LXR-α (liver X receptor alpha) were less consistent than other markers, suggesting complex feedback regulation. LDLR plays a key role in clearing LDL-C from circulation and is transcriptionally regulated by SREBP2 [6,38]. While some degree of downregulation was observed in the PN-treated groups, this may reflect a compensatory reduction following decreased circulating LDL-C levels, or suppression secondary to SREBP2 inhibition. LXR-α is known to upregulate CYP7A1 and ABCG5/8, promoting cholesterol efflux and bile acid production. Its downregulation in this study may be due to negative feedback from reduced cholesterol accumulation or altered ligand activation.

These results highlight the intricate network of cholesterol regulation involving multiple transcription factors, receptors, and feedback loops. Further studies focusing on protein localization, receptor activation states, and co-regulatory interactions may help clarify these observations.

### 4.6. Potential Microbiota–Bile Acid–Liver Axis Mediated Effects of Phytosterols

In addition to the direct modulation of hepatic cholesterol metabolism observed in this study, recent research suggests that phytosterols may exert broader systemic effects by interacting with the gut microbiota and influencing the enterohepatic circulation of bile acids [39]. Phytosterols can modulate the composition and metabolic activity of intestinal microbes, promoting the conversion of primary bile acids into secondary bile acids such as deoxycholic acid (DCA) and lithocholic acid (LCA) [40]. These microbial-derived bile acids serve as endogenous ligands for nuclear receptors such as FXR (farnesoid X receptor) and LXR (liver X receptor), which in turn regulate key hepatic genes involved in lipid homeostasis [41]. For example, FXR activation can repress CYP7A1 transcription via the FGF15–FGFR4 signaling axis, thereby altering bile acid synthesis and feedback inhibition [42]. Conversely, LXR activation has been shown to influence the expression of SREBP2 and LDLR, linking microbial metabolism to cholesterol uptake and biosynthesis [43].

Although our current study focused on hepatic HMGCR/SREBP2 inhibition and CYP7A1 upregulation at the protein level, it is plausible that part of this dual modulation may be mediated by microbiota-driven alterations in bile acid signaling. Future work will explore this hypothesis by quantifying changes in gut microbial composition, secondary bile acid profiles, and nuclear receptor activation to clarify the gut–liver mechanistic axis underlying nano-phytosterol function. It is worth noting that this study included only male mice, which may limit the generalizability of the findings across sexes. Future studies involving both male and female animals are warranted to fully evaluate sex-specific responses to nano-phytosterol intervention.

The cholesterol-lowering efficacy of PNs demonstrated in this high-fat diet-induced hypercholesterolemic mouse model suggests promising applications for both human and veterinary medicine. In humans, these nanoparticles could serve as a safe, plant-based nutraceutical alternative to pharmacological interventions like statins, particularly for individuals with statin intolerance or those preferring natural therapies, given the established safety profile of phytosterols in dietary use [29,30]. The dual modulation of cholesterol metabolism—via downregulation of HMGCR and SREBP2 to inhibit synthesis and upregulation of CYP7A1 to enhance catabolism—offers a multifaceted approach that could complement existing cholesterol management strategies. However, the enhanced bioavailability inferred from these superior cholesterol-lowering effects is limited by the absence of direct in vivo pharmacokinetic data, such as serum phytosterol concentrations or tissue distribution. Clinical trials are necessary to evaluate pharmacokinetics, optimal dosing, and long-term safety in humans. In veterinary medicine, phytosterol nanoparticles could be incorporated into functional feeds to manage lipid-related disorders in companion animals or enhance metabolic efficiency in livestock, though species-specific studies are needed to confirm efficacy and establish appropriate delivery systems. Future research will prioritize pharmacokinetic analyses to quantitatively assess absorption and bioavailability, strengthening the mechanistic insights and therapeutic potential of this approach.

## 5. Conclusions

This study demonstrated that PNs effectively ameliorate hypercholesterolemia in high-fat diet-induced mice through a multifaceted mechanism involving the inhibition of cholesterol synthesis, enhancement of cholesterol catabolism, and promotion of fecal cholesterol excretion. Specifically, the nanoparticles downregulated hepatic HMGCR and SREBP2 expression while upregulating CYP7A1 protein levels, thereby restoring lipid homeostasis. In addition to systemic improvements in serum and liver lipid profiles, histological analysis confirmed reduced hepatic steatosis. The increased fecal excretion of cholesterol and phytosterols further indicates inhibition of intestinal cholesterol absorption. Compared to simvastatin, PNs exhibited comparable efficacy with broader mechanistic coverage and greater potential for safe, long-term application. These findings suggest that PNs represent a promising nutraceutical intervention for managing dyslipidemia and associated metabolic disorders.

## Figures and Tables

**Table 1:** Experimental design.

Groups	Diets
Nor	standard diet
L-PN	HFD + 4.00 mg/mL of PN (0.5 mL)
M-PN	HFD + 8.25 mg/mL of PN (0.5 mL)
H-PN	HFD + 12.50 mg/mL of PN (0.5 mL)
HCM	HFD + 0.5 mL normal saline
PM	HFD + 0.40 mg/mL simvastatin (0.5 mL)

**Table 2:** Composition and nutrient levels of experimental diets (dry matter basis).

Ingredient	Nor	HFD
Casein	200	200
DL-Methionine	3	3
Corn Starch	600	410
Maltodextrin	50	50
Cellulose	50	50
Sucrose	1	1
Lard	45	225
Cholesterol	0	10
Mineral Mix AIN93	23.6	23.6
Vitamin Mix AIN93	14.4	14.4
Choline	1	1
Potassium Citrate	10	10
NaCl	2	2
Fat (%, *w*/*w*)	10	45
Energy (kcal/g diet)	3.87	4.69

**Table 3:** Primer sequence and product size.

Primer Name	Sequence (5′-3′)	Amplified Fragment Size (bp)
SREBP2	F: CAGACCTAGACCTCGC	244
	R: CTGCTCTTAGCCTCAT	
LDLR	F: GTCTGCTCCCCAACCT	264
	R: TCCTCCTCTTTACGCC	
HMGCR	F: AATAAACCAAACCCCG	264
	R: GACAGCCAAAAGGAAG	
CYP7A1	F: GAGAAGGAAAGTAGGTGAA	174
	R: AGGGAGTTTGTGATGAAG	
LXRa	F: ATGTTTCTCCTGATTCTGC	140
	R: CTCCAACCCTATCCCTAA	
β Actin	F: CCCATCTACGAGGGCTAT	145
	R: TGTCACGCACGATTTCC	

**Table 4:** NAFLD Activity Scores for Liver Histology Across Experimental groups.

Group	Steatosis (0–3)	Inflammation (0–3)	Ballooning (0–2)	Total NAS (0–8)
Nor	0	0	0	0
HCM	3	2	2	7
PM	1	1	1	3
L-PN	2	1	1	4
M-PN	1	1	1	3
H-PN	1	0–1	0–1	2–3

**Table 5:** Fecal cholesterol and sterol contents.

Testing Index	Nor	HCM	L-PN	M-PN	H-PN	PM
Cholesterol content (mg/g feces)	1.26 ± 0.03 ^A^	11.79 ± 0.24 ^B^	28.80 ± 0.17 ^C^	32.73 ± 0.34 ^D^	36.01 ± 0.34 ^E^	36.90 ± 0.24 ^F^
Sterol content (mg/g feces)	---	---	6.90 ± 0.14	10.04 ± 0.05	13.07 ± 0.08	---

“---” indicates that sterols were not detected. Different capital letters indicate significant differences between groups, *p* < 0.01. Nor: fed basic feed + 0.5 mL saline gavage daily; HCM: fed high-fat feed + 0.5 mL saline gavage daily; L-PN: fed high-fat feed + 0.5 mL low concentration (4.00 mg/mL) PNs by gavage daily; M-PN: fed high-fat feed + 0.5 mL medium concentration (8.25 mg/mL) PNs by gavage daily; H-PN: fed high-fat diet + 0.5 mL high concentration (12.50 mg/mL) PNs by daily gavage; PM: fed high-fat diet + 0.5 mL 0.4 mg/mL simvastatin by daily gavage. *n* = 10.

## Data Availability

The original contributions presented in this study are included in the article. Further inquiries can be directed to the corresponding author.

## References

[1] Frampton J.E. (2023). Inclisiran: A Review in Hypercholesterolemia. Am. J. Cardiovasc. Drugs.

[2] Mormone A., Tortorella G., Esposito F., Caturano A., Marrone A., Cozzolino D., Galiero R., Marfella R., Sasso F.C., Rinaldi L. (2024). Advances in Pharmacological Approaches for Managing Hypercholesterolemia: A Comprehensive Overview of Novel Treatments. Biomedicines.

[3] Pećin I., Reiner Ž. (2021). Novel experimental agents for the treatment of hypercholesterolemia. J. Exp. Pharmacol..

[4] Turini E., Sarsale M., Petri D., Totaro M., Lucenteforte E., Tavoschi L., Baggiani A. (2022). Efficacy of Plant Sterol-Enriched Food for Primary Prevention and Treatment of Hypercholesterolemia: A Systematic Literature Review. Foods.

[5] Feng S., Wang L., Shao P., Sun P., Yang C.S. (2022). A review on chemical and physical modifications of phytosterols and their influence on bioavailability and safety. Crit Rev Food Sci Nutr..

[6] Li X., Xin Y., Mo Y., Marozik P., He T., Guo H. (2022). The bioavailability and biological activities of phytosterols as modulators of cholesterol metabolism. Molecules.

[7] Pandak W.M., Schwarz C., Hylemon P.B., Mallonee D., Valerie K., Heuman D.M., Fisher R.A., Redford K., Vlahcevic Z.R. (2001). Effects of CYP7A1 overexpression on cholesterol and bile acid homeostasis. Am. J. Physiol.—Gastrointest. Liver Physiol..

[8] Zhang Z.S., Hou D.M., He W., Wang J.Y. (2011). Effect of phytosterol on blood lipid in hyperlipidemia rats. Food Sci..

[9] Sharpe L.J., Brown A.J. (2013). Controlling cholesterol synthesis beyond 3-hydroxy-3-methylglutaryl-CoA reductase (HMGCR). J. Biol. Chem..

[10] Li A., Zhu A., Kong D., Wang C., Liu S., Zhou L., Cheng M. (2022). Water-dispersible phytosterol nanoparticles: Preparation, characterization, and in vitro digestion. Front. Nutr..

[11] Zhang J., Pan W., Wang Y., Zhang C., Wang C., Li S., Chen F., Zhu A. (2024). Enhancing vaccine efficacy: Evaluating the superiority of cationic liposome-embedded squalene adjuvant against PCV2 infection. Virology.

[12] Kuang M., Yu H., Qiao S., Huang T., Zhang J., Sun M., Shi X., Chen H. (2021). A Novel Nano-Antimicrobial Polymer Engineered with Chitosan Nanoparticles and Bioactive Peptides as Promising Food Biopreservative Effective against Foodborne Pathogen *E. coli* O157-Caused Epithelial Barrier Dysfunction and Inflammatory Responses. Int. J. Mol. Sci..

[13] Yu H.T., Zhang J.Q., Sun M.C., Zhao Y., Wang L., Chen X. (2022). Polymeric Nanohybrids Engineered by Chitosan Nanoparticles and Antimicrobial Peptides as Novel Antimicrobials in Food Biopreservatives: Risk Assessment and Anti-Foodborne Pathogen. J. Agric. Food Chem..

[14] Kleiner D.E., Brunt E.M., Van Natta M., Behling C., Contos M.J., Cummings O.W., Ferrell L.D., Liu Y.C., Torbenson M.S., Unalp-Arida A. (2005). Design and Validation of a Histological Scoring System for Nonalcoholic Fatty Liver Disease. Hepatology.

[15] Hayat A., Aal A.E., Lamiaa A., El-Mahdy M.M. (2017). Combination of Carvacrol with Simvastatin Improves the Lipid-Lowering Efficacy and Alleviates Simvastatin Side Effects. J. Biochem. Mol. Toxicol..

[16] Hu N., Chen C., Wang J., Huang J., Yao D., Li C. (2021). Atorvastatin Ester Regulates Lipid Metabolism in Hyperlipidemia Rats via the PPAR-signaling Pathway and HMGCR Expression in the Liver. Int. J. Mol. Sci..

[17] Horton J.D., Goldstein J.L., Brown M.S. (2002). SREBPs: Activators of the complete program of cholesterol and fatty acid synthesis in the liver. J. Clin. Investig..

[18] Benatzy Y., Palmer M.A., Lütjohann D., Ohno R., Kampschulte N., Schebb N.H., Fuhrmann D.C., Snodgrass R.G., Brüne B. (2024). ALOX15B controls macrophage cholesterol homeostasis via lipid peroxidation, ERK1/2 and SREBP2. Redox. Biol..

[19] Xu M., Jiang S., Tang S., Zhu M., Hu Y., Li J., Yan J., Qin C., Tan D., An Y. (2025). Nuclear SREBP2 condensates regulate the transcriptional activation of lipogenic genes and cholesterol homeostasis. Nat. Metab..

[20] De Fabiani E., Mitro N., Anzulovich A.C., Pinelli A., Galli G., Crestani M. (2001). The negative effects of bile acids and tumor necrosis factor-α on the transcription of cholesterol 7α-hydroxylase gene (CYP7A1) converge to hepatic nuclear factor-4: A novel mechanism of feedback regulation of bile acid synthesis mediated by nuclear receptors. J. Biol. Chem..

[21] Tempel W., Grabovec I., MacKenzie F., Dichenko Y.V., Usanov S.A., Gilep A.A., Park H., Strushkevich N. (2014). Structural characterization of human cholesterol 7α-hydroxylase. J. Lipid Res..

[22] Song K.H., Li T., Owsley E., Chiang J. (2010). A putative role of micro RNA in regulation of cholesterol 7α-hydroxylase expression in human hepatocytes. J. Lipid Res..

[23] Chun K.-H. (2022). Molecular Targets and Signaling Pathways of microRNA-122 in Hepatocellular Carcinoma. Pharmaceutics.

[24] Jang S., Lee M.-S., Kang S.-A., Kim C.-T., Kim Y. (2022). Portulaca oleracea L. Extract Regulates Hepatic Cholesterol Metabolism via the AMPK/MicroRNA-33/34a Pathway in Rats Fed a High-Cholesterol Diet. Nutrients.

[25] Wang M., Tan Y., Costa R.H., Holterman A.X.L. (2004). In vivo regulation of murine CYP7A1 by HNF-6: A novel mechanism for diminished CYP7A1 expression in biliary obstruction. Hepatology.

[26] Chen Z., Ding H., Zhu H., Huang S., Yan C., Chen Z. (2024). Additional mechanism for selective absorption of cholesterol and phytosterols. Food Chem..

[27] Stellaard F., Lütjohann D. (2025). Phytosterol-Enriched Dietary Supplements for Lowering Plasma LDL-Cholesterol: Yes or No?. Nutrients.

[28] Zhang R., Han Y., McClements D.J., Xu D., Chen S. (2022). Production, characterization, delivery, and cholesterol-lowering mechanism of phytosterols: A review. J. Agric. Food Chem..

[29] Eng J.M., Estall J.L. (2021). Diet-induced models of non-alcoholic fatty liver disease: Food for thought on sugar, fat, and cholesterol. Cells.

[30] Li H., Yu X., Ou X., Ouyang X., Tang C. (2021). Hepatic cholesterol transport and its role in non-alcoholic fatty liver disease and atherosclerosis. Prog. Lipid Res..

[31] Poudel P., Petropoulos S.A., Di Gioia F. (2023). Plant Tocopherols and Phytosterols and Their Bioactive Properties. Natural Secondary Metabolites: From Nature, Through Science, to Industry.

[32] Shen M., Yuan L., Zhang J., Wang X., Zhang M., Li H., Jing Y., Zeng F., Xie J. (2024). Phytosterols: Physiological Functions and Potential Application. Foods.

[33] Al-Kuraishy H.M., Al-Gareeb A.I., Alexiou A., Papadakis M., Alsayegh A.A., Almohmadi N.H., Saad H.M., Batiha G.E.S. (2023). Pros and cons for statins use and risk of Parkinson’s disease: An updated perspective. Pharmacol. Res. Perspect..

[34] Sattar N. (2023). Statins and diabetes: What are the connections?. Best Pract. Res. Clin. Endocrinol. Metab..

[35] Micallef M.A., Garg M.L. (2009). Beyond blood lipids: Phytosterols, statins and omega-3 polyunsaturated fatty acid therapy for hyperlipidemia. J. Nutr. Biochem..

[36] Du Y., Xi M., Li Y., Zheng R., Ding X., Li X., Zhang X., Wang L., Xing J., Hong B. (2023). Tilianin improves lipid profile and alleviates atherosclerosis in ApoE^−/−^ mice through up-regulation of SREBP2-mediated LDLR expression. Phytomedicine.

[37] Li Y., Song Y., Zhao M., Guo Y., Yu C., Chen W., Shao S., Xu C., Zhou X., Zhao L. (2017). A novel role for CRTC2 in hepatic cholesterol synthesis through SREBP-2. Hepatology.

[38] Davis H.R., Zhu L., Hoos L.M., Tetzloff G., Maguire M., Liu J., Yao X.R., Iyer S.P., Lam M., Lund E.G. (2004). Niemann-Pick C1 Like 1 (NPC1L1) is the intestinal phytosterol and cholesterol transporter and a key modulator of whole-body cholesterol homeostasis. J. Biol. Chem..

[39] Lv W.J., Huang J.Y., Lin J., Wang Z., Fang H., Zhu X., Liu J., Cao Y., Wang F., Zhang Y. (2023). Phytosterols Alleviate Hyperlipidemia by Regulating Gut Microbiota and Cholesterol Metabolism in Mice. Oxid. Med. Cell. Longev..

[40] Zhang Y., Gu Y., Jiang J., Shen H., Tian L., Liu S., Jiang Y. (2022). Stigmasterol Attenuates Hepatic Steatosis in Rats by Strengthening the Intestinal Barrier and Improving Bile Acid Metabolism. NPJ Sci. Food.

[41] Qin D., Pan P., Lyu B., Zhang R., Yang Y., Su J., Pan W., Wei L. (2024). Lupeol Improves Bile Acid Metabolism and Metabolic Dysfunction-Associated Steatotic Liver Disease in Mice via FXR Signaling Pathway and Gut-Liver Axis. Biomed. Pharmacother..

[42] Katafuchi T., Makishima M. (2022). Molecular Basis of Bile Acid-FXR-FGF15/19 Signaling Axis. Int. J. Mol. Sci..

[43] Cao X., Fang W., Li X., Chen Y., Lu Q., Zhou Y., Chi S., Tan B., Zhang H., Dong X. (2022). Increased LDL Receptor by SREBP2 or SREBP2-Induced lncRNA LDLR-AS Promotes Triglyceride Accumulation in Fish. iScience.

